# My friend, my companion, my social sidekick! Social anxiety and tobacco smoking

**DOI:** 10.1192/j.eurpsy.2023.1377

**Published:** 2023-07-19

**Authors:** I. Ganhao, A. Paixao

**Affiliations:** Centro Hospitalar Psiquiátrico de Lisboa, Lisbon, Portugal

## Abstract

**Introduction:**

In mental health settings, there is no place more social than where people smoke tobacco, patients and healthcare professionals alike much as many social activities in other settings even nowadays.

Yet mental illness is associated with higher levels of social anxiety. Those who suffer are doing their coping and may appear to be doing better than the others but in fact may need special attention for smoking cessation because they are still smoking more than other patient populations.

**Objectives:**

To reflect on tobacco smoking and social anxiety.

**Methods:**

Pubmed search using terms: tobacco and smoking and social anxiety/ social anxiety disorder

**Results:**

Social anxiety:

1. Is associated with higher smoking initiation and progression to dependence
2. is more frequent in smokers
3. is used as a coping mechanism for distress caused by social interactions and may alleviate negative affect and thus serve as negative reinforcement
4. may be associated with higher nicotine dependence
5. has not been definitely associated with heavier smoking
6. may differ in its effects according to gender
7. may be associated with less quit attempts
8. may hinder success in quitting smoking and may be associated with higher rates of relapse

**Conclusions:**

Identifying and treating social anxiety may lead to better outcomes in smoking cessation in a sub-group of patients who present elevated social anxiety with or without social anxiety disorder.

Patients with mental illness, especially serious mental illness, will likely present with higher levels of social anxiety which may represent a significant factor contributing to an increased difficulty in quitting tobacco smoking in this patient population.

**Disclosure of Interest:**

None Declared

